# Supplementary material for: Glycol Chitosan-Docosahexaenoic Acid Liposomes for Drug Delivery: Synergistic Effect of Doxorubicin-Rapamycin in Drug-Resistant Breast Cancer
Source: Mar Drugs. 2019 Oct 12;17(10):581. doi: 10.3390/md17100581 (PMC6835303; doi:10.3390/md17100581)
Supplement: Supplementary file 1 [file marinedrugs-17-00581-s001.pdf]

Supplementary Information

Article

Glycol chitosan-docosahexaenoic acid liposomes for drug delivery: synergistic effect of doxorubicin-rapamycin in drug-resistant breast cancer

Min Woo Kim [1], Takuro Niidome [2], and Ruda Lee [1*]

[1] International Research Organization for Advance Science and Technology (IROAST), Kumamoto University, Kumamoto, Japan; minwoo-kim@kumamoto-u.ac.jp

[2] Faculty of Advanced Science and Technology, Kumamoto University, Kumamoto, Japan; niidome@kumamoto-u.ac.jp

[*] Correspondence: aeju-lee@kumamoto-u.ac.jp

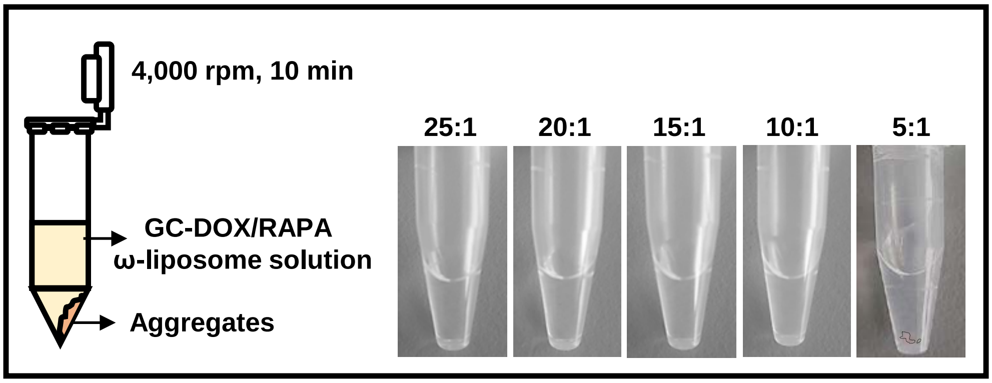

Figure S1. The images of GC-DOX/RAPA ω-liposome sample at different ratios after centrifugation. Precipitate (dotted line) stands for the aggregated GC-DOX/RAPA ω-liposomes in a tube after centrifugation. Supernatant is the remaining GC-DOX/RAPA ω-liposome solution.

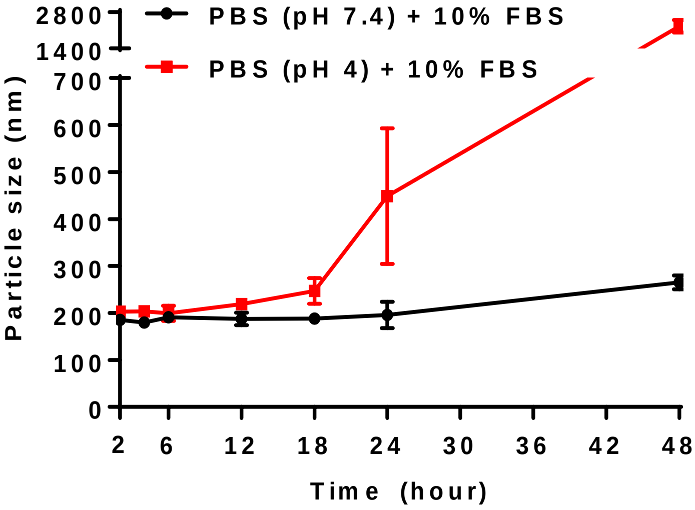

Figure S2. Colloidal stability of GC-DOX/RAPA ω-liposomes at pH 7.4 and pH 4 in 10% FBS. Particle size was monitored with dynamic light scattering over 48 hours (n = 3/time point).

Formulations	IC_{50}RAPA (nM)	IC_{50}DOX (nM)	Combination Index (CI)
**RAPA ω-liposomes**	74.12	N/A	N/A
**GC-DOX**	N/A	856.10	N/A
**GC-DOX/RAPA ω-liposomes (1:1.5)**	12.24	8.16	0.18

CI<1 Synergy
CI=1 Additive
CI>1 Antagonism

$$\text{Combination Index (CI)} = \frac{C_A}{IC_{50A}} + \frac{C_B}{IC_{50B}}$$

Figure S3. Drug combination index analysis for selected DOX and RAPA ratio (1:1.5) in MDA-MB-231-GFP/DOX cell lines. For drug combination testing, the cells were exposed to the indicated drugs for 72 h, and the IC_{50} and combination index (CI) was calculated according to the equation.

Table S1. Components of GC-DOX/RAPA ω-liposomes

Formulations	Lipid composition (2 mg/mL)			Drug composition		E.E.		L.E.	
	DPPC	CHEMS	DHA	RAPA	DOX	RAPA	DOX	RAPA	DOX
**RAPA ω-liposomes**	47.5 mol%	47.5 mol%	5 mol%	10:1 (w/w)	-	69.88%	-	6.27%	-
**GC-DOX/RAPA ω-liposomes**	47.5 mol%	47.5 mol%	5 mol%	10:1 (w/w)	20:1 (w/w)	69.88%	90.29%	6.27%	4.05%
